# Mitochondria damaged by Oxygen Glucose Deprivation can be Restored through Activation of the PI3K/Akt Pathway and Inhibition of Calcium Influx by Amlodipine Camsylate

**DOI:** 10.1038/s41598-019-52083-y

**Published:** 2019-10-31

**Authors:** Hyun-Hee Park, Myung-Hoon Han, Hojin Choi, Young Joo Lee, Jae Min Kim, Jin Hwan Cheong, Je Il Ryu, Kyu-Yong Lee, Seong-Ho Koh

**Affiliations:** 1 Departments of Neurology, Hanyang University Guri Hospital, 11923 Guri, Korea; 2 Departments of Neurosurgery, Hanyang University Guri Hospital, 11923 Guri, Korea; 3 Department of Translational Medicine, Hanyang University Graduate School of Biomedical Science & Engineering, 04763 Seoul, Korea

**Keywords:** Neurological disorders, Molecular neuroscience

## Abstract

Amlodipine, a L-type calcium channel blocker, has been reported to have a neuroprotective effect in brain ischemia. Mitochondrial calcium overload leads to apoptosis of cells in neurologic diseases. We evaluated the neuroprotective effects of amlodipine camsylate (AC) on neural stem cells (NSCs) injured by oxygen glucose deprivation (OGD) with a focus on mitochondrial structure and function. NSCs were isolated from rodent embryonic brains. Effects of AC on cell viability, proliferation, level of free radicals, and expression of intracellular signaling proteins were assessed in OGD-injured NSCs. We also investigated the effect of AC on mitochondrial structure in NSCs under OGD by transmission electron microscopy. AC increased the viability and proliferation of NSCs. This beneficial effect of AC was achieved by strong protection of mitochondria. AC markedly enhanced the expression of mitochondrial biogenesis-related proteins and mitochondrial anti-apoptosis proteins. Together, our results indicate that AC protects OGD-injured NSCs by protecting mitochondrial structure and function. The results of the present study provide insight into the mechanisms underlying the protective effects of AC on NSCs.

## Introduction

Oxygen gluco se deprivation (OGD) leading to free radical attack against neural cells contributes to neuronal cell death^1^. Hypertension is a major risk factor for ischemic stroke and is associated with OGD in the brain^2^. A long acting L-type calcium channel blocker, amlodipine, is one of the drugs most commonly used to treat hypertension^3,4^. Amlodipine has been reported to have a neuroprotective effect in the ischemic brain^5–11^. Previous studies have shown that amlodipine reduces stroke size, oxidative stress, enhances survival signals, and inhibits death signals^5–11^. However, to our knowledge, there are no published studies regarding the association between amlodipine and mitochondrial ultrastructure and function in neural stem cells (NSCs). In this study, instead of neuronal cultures or brain slices, we have used NSCs to investigate the association between amlodipine and NSCs injured by OGD; this could help in predicting the effect of amlodipine on regeneration and recovery after stroke^12^.

It is well documented that mitochondrial calcium overload induces activation of the mitochondrial permeability transition (MPT) and that this process leads to loss of mitochondrial membrane potential, swelling of the mitochondrial matrix, and outer membrane rupture, followed by cytochrome *c* release, activation of downstream cascades, and ultimately apoptosis of cells in neurologic diseases^13,14^. In light of this knowledge, we hypothesized that amlodipine, via its calcium inhibitory properties, would reduce both cellular and mitochondrial calcium levels and subsequently inhibit activation of the MPT, and that this process would help maintain mitochondrial membrane potential and structure with reduction of cytochrome *c* release, leading to protection of NSCs from OGD.

In the present study, we evaluated the neuroprotective effects of amlodipine camsylate (AC) on NSCs exposed to OGD with a focus on mitochondrial structure and function. We also examined the effects of AC on intracellular survival signaling proteins and mitochondrial biogenesis-related proteins in NSCs.

## Results

### Effects of OGD and AC on the viability and toxicity of NSCs

**Figure 1:**
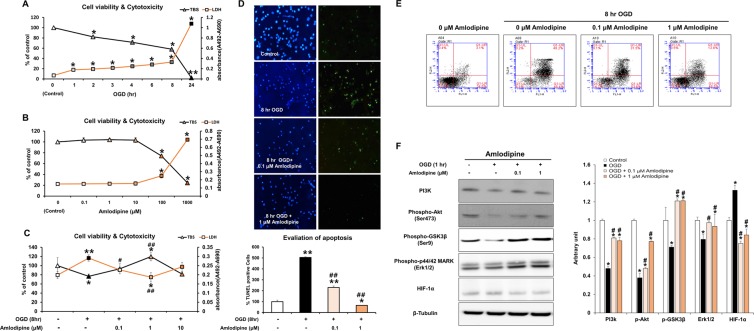
Effect of OGD and AC on viability of neural stem cells. (**A**) Neural stem cells (NSCs) were treated with oxygen–glucose deprivation (OGD) for different periods of time. OGD induced cytotoxicity and decreased the viability of NSCs in a time-dependent manner. (**B**) To evaluate the effect of AC itself on NSCs, cells were treated with several concentrations of AC (0, 0.1, 1, 10, 100, or 1000 μM) for 8 h. AC (up to 10 μM) did not affect viability and was not cytotoxic to NSCs. (**C**) To measure the effects of AC on OGD-induced neurotoxicity, NSCs were simultaneously treated with several concentrations of AC (0, 0.1, 1, or 10 μM) and OGD for 8 h. AC up to 1 μM protected NSCs from OGD, but 10 μM AC was not protective. DAPI and TUNEL staining (**D**) and Annexin V/PI FACS analysis (**E**) showed that AC effectively blocked apoptotic cell death of NSCs induced by OGD. (**F**) AC restored the levels of each of these signaling proteins in NSCs under OGD. Data are means (% of control) ± SD from five independent experiments. Treatment groups were compared with the control group using Tukey’s test after one-way ANOVA. *p < 0.05 and **p < 0.01 (vs. control group), ^#^p < 0.05 and ^##^p < 0.05 (vs. the group treated only with OGD). Full-length blots/gels are presented in Supplementary Fig. S1.

To measure the alteration in neural stem cell (NSC) viability after exposure to OGD, NSCs were incubated with OGD for different exposure times. Cell viability and death were measured with trypan blue staining (TBS) (0, 2, 4, 8, and 24 hours) and LDH assay (0,1,2,3,4,6,8, and 24 hours); results are shown in Fig. 1A. A significant decrease in NSC viability and increase in cell death were observed after exposure to hypoxic conditions in a time-dependent manner. The cytotoxicity of AC was evaluated by treating cells with different concentrations of AC (Fig. 1B). Treatment of cells with above 100 μM AC significantly reduced NSC viability. To identify the effect of AC on cell viability in the context of OGD, we co-treated cells with OGD and three different concentrations of AC for 8 hours (0.1,1 and 10 μM) (Fig. 1C). Cells treated with 1 μΜ AC under OGD for 8 hours showed significantly higher NSC viability and lower NSC toxicity than the cells treated with the other two concentrations of AC and OGD. Treatment of cells with 10 μΜ AC decreased the viability of NSCs exposed to OGD.

### Anti-apoptotic effect of AC on NSCs under OGD

TUNEL and DAPI staining were performed to examine the effect of AC on OGD-induced apoptosis of NSCs (Fig. 1D). Percentage of apoptotic cells increased significantly after treatment with OGD for 8 hours. However, the percentage apoptotic cells decreased significantly upon treatment with AC in a concentration-dependent manner. We also measured the apoptosis of NSCs using fluorescence-activated cell sorting (FACS) and observed a significant reduction in the percentage of apoptotic cells upon treatment with 1 μΜ AC under OGD for 8 hours (Fig. 1E).

### Effect of AC on NSC intracellular signaling

To investigate the effects of AC on signaling proteins associated with the survival of NSCs, we analyzed PI3K, phospho-Akt (Ser473), phospho-GSK3β (Ser9), and phospho-p44/42 MARK (Erk1/2) expression by western blotting (Fig. 1F). Immunoreactivities of PI3K, phospho-Akt (Ser473), phospho-GSK3β (Ser9), and phospho-p44/42 MARK (Erk1/2) were higher in NSCs co-treated with AC than in NSCs treated with OGD alone. To determine whether NSCs were affected by OGD, we also measured levels of HIF-1α in the NSCs by western blotting. HIF-1α expression was significantly increased in NSCs treated with OGD alone, but it was markedly decreased in NSCs co-treated with AC and OGD.

### Effect of AC on NSC proliferation under OGD

**Figure 2:**
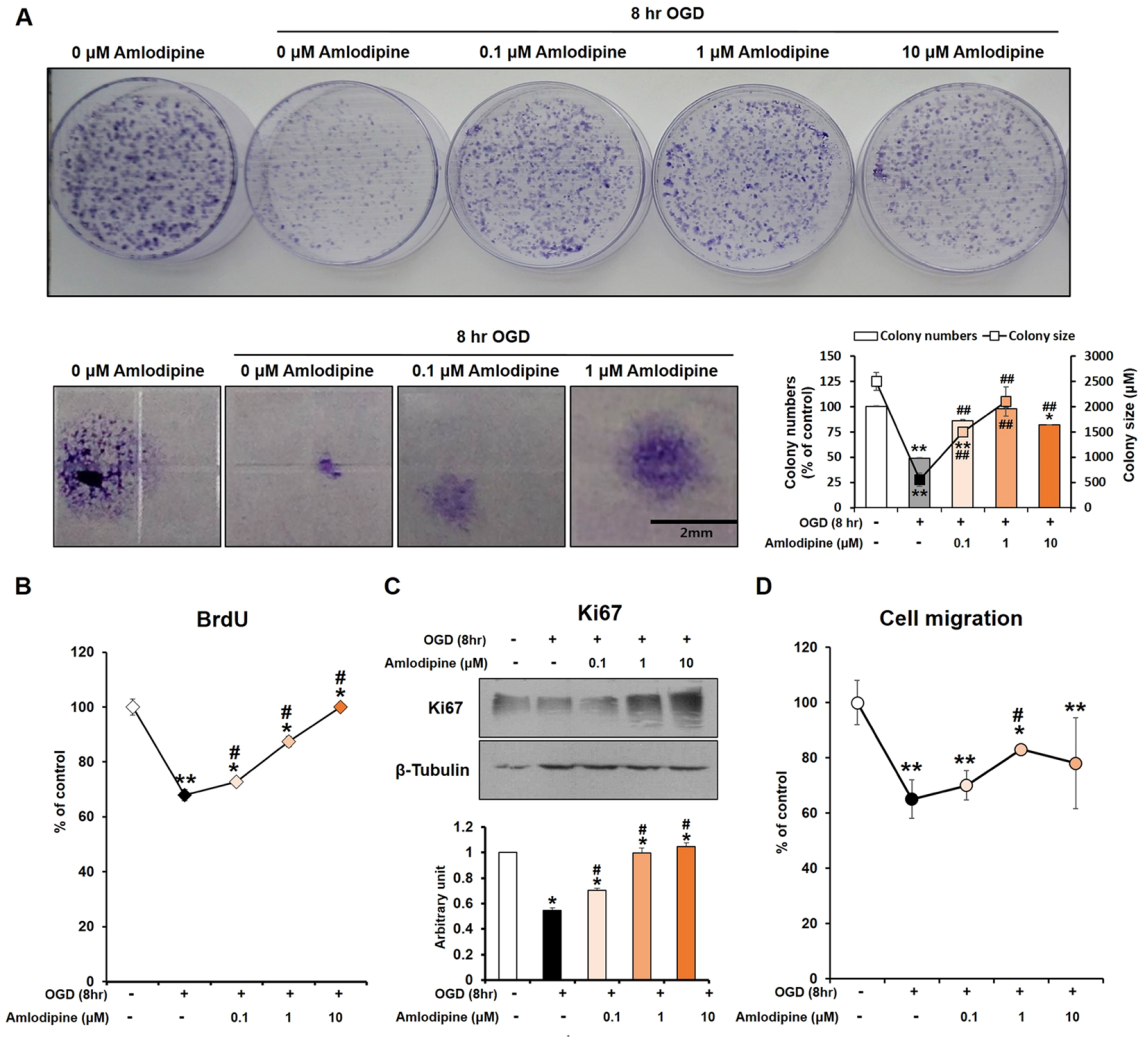
Effect of OGD and AC on NSC proliferation. (**A**) Colony forming unit (CFU) assays were performed. The OGD-induced decrease in proliferative activity of NSCs was significantly recovered by treatment with AC. BrdU assay (**B**) and Ki67 staining (**C**) also showed that AC effectively increased proliferation of NSCs subjected to OGD. (**D**) A cell migration assay showed that the migratory activity of NSCs was reduced by OGD but strongly increased by AC treatment. Data are means (% of control) ± SD from five independent experiments. Treatment groups were compared with the control group using Tukey’s test after one-way ANOVA. *p < 0.05 and **p < 0.01 (vs. control group), ^#^p < 0.05 (vs. the group treated only with OGD). Full-length blots/gels are presented in Supplementary Fig. S2.

NSC proliferation was assessed by colony formation assay. Compared with NSCs treated with OGD alone for 8 hours, co-treatment with AC significantly increased NSC proliferation in a concentration-dependent manner (Fig. 2A,B). In addition, Ki67 immunoreactivity (proliferation marker) was increased in NSCs co-treated with AC compared to cells treated with OGD alone (Fig. 2C). Furthermore, NSCs treated with AC under OGD showed significantly higher cell migration activities than NSCs treated with OGD alone for 8 hours (Fig. 2D).

### Protective effect of AC on mitochondrial cristae ultrastructure and function in NSCs injured by OGD

**Figure 3:**
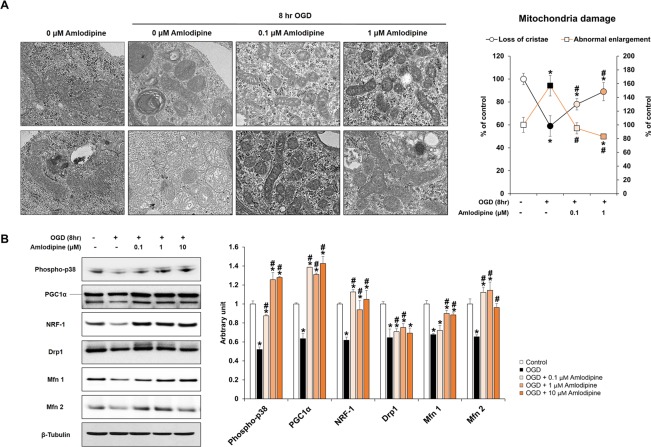
Effect of OGD and AC on NSC mitochondria. (**A**) Representative transmission electron microscopy (TEM) images of NSCs treated with OGD only (left) or OGD and AC (right). Mitochondrial damage was characterized by the loss of cristae (top) and abnormal enlargement (bottom). OGD-induced mitochondrial damage was prevented by AC treatment. (**B**) Western blot showing that expression levels of proteins associated with mitochondria biogenesis of NSCs, such as phospho-p38, PGC1α, NRF-1, Drp1, Mfn 1, and Mfn 2, were decreased by OGD treatment but increased with AC treatment. Data are means (% of control) ± SD from five independent experiments. Treatment groups were compared with the control group using Tukey’s test after one-way ANOVA. ^*^p < 0.05 (vs. control group), ^#^p < 0.05 (vs. the group treated only with OGD). Full-length blots/gels are presented in Supplementary Fig. S4.

Transmission electron microscopy (TEM) was used to evaluate ultrastructural changes of the cristae in the mitochondria of NSCs subjected to OGD and/or AC treatment (Fig. 3A). Structural enlargement of the mitochondrial matrix and deformation of the mitochondrial cristae were observed in NSCs treated with OGD for 8 hours compared with the control. However, co-treatment of NSCs with AC plus OGD for 8 hours resulted in significant preservation of mitochondrial cristae ultrastructure in a concentration-dependent manner. Low magnification TEM images are presented in Supplementary Fig. S3. In addition, mitochondrial function was evaluated in NSCs treated with OGD and/or AC by western blotting using mitochondrial biogenesis-related marker antibodies (Fig. 3B). Significantly higher expression of mitochondrial biogenesis-related proteins was observed in NSCs co-treated with AC and OGD than in cells treated with OGD alone.

### Effect of AC on OGD-induced free radical production and mitochondrial damage in NSCs

**Figure 4:**
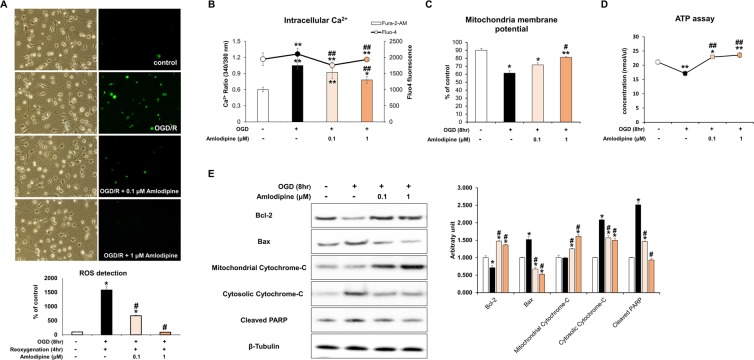
Effect of AC on mitochondrial oxidative stress in NSCs injured by OGD. (**A**) NSCs were treated with OGD for 30 m and free radical generation was measured using the fluorescent probe DCFH-DA. OGD significantly increased levels of intracellular reactive oxygen species (ROS). However, co-treatment with AC dramatically decreased ROS levels in NSCs. (**B**) Measurement of intracellular Ca^2+^ levels suggested that intracellular Ca^2+^ influx into NSCs was increased by OGD but is decreased by AC treatment. (**C**) Mitochondrial membrane potential was measured in NSCs under diverse conditions and it decreased significantly upon OGD treatment, whereas AC effectively restored membrane potential to almost normal. (**D**) ATP level decreased with OGD while AC increased ATP level. (**E**) Western blots show that expression of proteins associated with cell survival, such as Bcl-2 and mitochondrial cytochrome *c*, decreased in response to OGD treatment but increased with AC treatment. Proteins related to cell death, including Bax, cytosolic cytochrome *c*, and cleaved PARP increased with OGD treatment but decreased with AC treatment. Data are means (% of control) ± SD from five independent experiments. Treatment groups were compared with the control group using Tukey’s test after one-way ANOVA. *p < 0.05 and **p < 0.01 (vs. control group), ^#^p < 0.05 (vs. the group treated only with OGD). Full-length blots/gels are presented in Supplementary Fig. S6.

Oxidative damage was evaluated by detecting production of reactive oxygen species (ROS) in NSCs. NSCs were treated with re-oxygenation for 4 hours following OGD for 8 hours and ROS levels were assessed using DCF fluorescence (Fig. 4A). Treatment with OGD for 8 hours followed by re-oxygenation for 4 hours resulted in significantly increased free radical production in NSCs compared with the control group. However, co-treatment with AC (0.1 and 1 μM) resulted in a significant decrease in ROS production in a concentration-dependent manner. Additional analyses were conducted to evaluate mitochondrial changes in NSCs treated with OGD and/or AC. Intracellular calcium level and mitochondrial membrane potential were measured, and an ATP assay was performed. Intracellular calcium levels were significantly lower in NSCs co-treated with AC than in cells treated with OGD alone (Fig. 4B). In addition, mitochondrial membrane potential and ATP concentrations were significantly higher in NSCs co-treated with AC and OGD than cells treated with OGD alone (Fig. 4C,D). Using FACS, lower JC-1 monomer expression was also confirmed in NSCs co-treated with AC compared to NSCs treated with OGD alone (Supplementary Fig. S5).

### Effect of AC on mitochondrial apoptosis-related proteins in NSCs

The effect of AC on mitochondrial apoptosis-related proteins was evaluated in NSCs that were injured by OGD using western blotting (Fig. 4E). Expression of the survival-related protein Bcl-2 and mitochondrial cytochrome *c* was significantly increased in NSCs co-treated with AC than NSCs treated with OGD alone. However, the expression of the apoptosis-related protein Bax, cytosolic cytochrome *c*, and cleaved PARP decreased in response to co-treatment with AC compared to cells treated with OGD alone.

### The PI3K/Akt pathways play a critical role in mediating AC-induced protective effects and restoration of mitochondrial damage in NSCs injured by OGD

**Figure 5:**
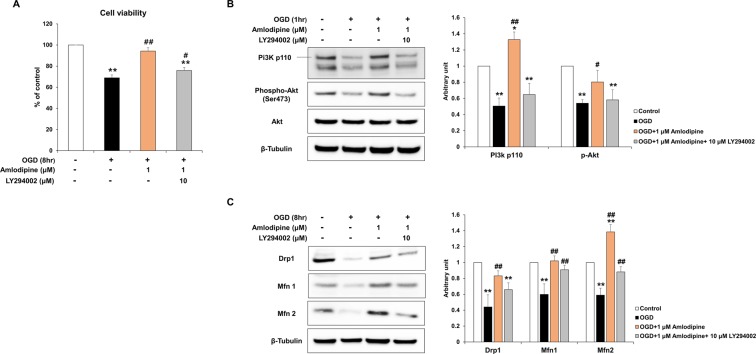
Role of the PI3K/Akt pathway in mediating the protective effect of AC in NSCs injured by OGD. (**A**) NSCs were pre-treated with LY294002, a PI3K inhibitor, (10 µM) and the graph shows significant inhibition of the protective effect of AC on NSCs injured using OGD. (**B**) Western blots show that expression of the PI3K/Akt pathway associated proteins increased in response to AC treatment in NSCs injured using OGD, and treatment with LY294002 decreased expression. (**C**) LY294002 also inhibited the effect of amlodipine on the expression of the mitochondrial structure and function-related proteins such as Drp1, Mfn1, and Mfn2 in NSCs injured using OGD. Data are means (% of control) ± SD from five independent experiments. Treatment groups were compared with the control group using Tukey’s test after one-way ANOVA. *p < 0.05 and **p < 0.01 (vs. control group), ^#^p < 0.05 and ^##^p < 0.01 (vs. the group treated only with OGD). Full-length blots/gels are presented in Supplementary Figs S7 and S8.

The roles of the PI3K/Akt pathways in mediating the protective effect of AC on the NSCs were investigated. NSCs were pre-treated with LY294002, a PI3K inhibitor, before OGD and/or AC treatment. LY294002 blocked the protective effect of AC on NSCs damaged by OGD, and cell viability was decreased by approximately 18%, compared to NSCs that were not pre-treated with the PI3K inhibitor (Fig. 5A). The levels of the PI3k/Akt pathway related signaling proteins, and mitochondrial structure and function related proteins, were also measured. The expression of PI3K p110, phospho-Akt (Ser473), Drp1, Mfn1 and Mfn2 was significantly decreased in NSCs pre-treated with LY294002 (Fig. 5B,C).

## Discussion

Previously, we reported that amlodipine had possible neuroprotective effects on oxidative stress-injured cortical neurons and NSCs^5,6^. In the present study, we focused on the effects of AC on mitochondrial structure and function in OGD-injured NSCs. AC increased cell viability, PI3K survival signaling, and NSC proliferation (Figs 1 and 2). AC also effectively preserved the mitochondrial structure of NSCs based on TEM findings (Fig. 3A). In addition, AC enhanced the expression of mitochondrial biogenesis-related proteins (Fig. 3B) and inhibited the expression of mitochondrial anti-apoptosis proteins (Fig. 4). Based on these findings, we hypothesize that AC improves cell survival by protecting mitochondria and inhibiting apoptosis in OGD-injured NSCs. Although previous studies have reported possible neuroprotective effects of amlodipine, this study is the first to identify the protective effects of AC on mitochondria in OGD-injured NSCs^7,8,10,11^. In addition, we identified that the PI3K/Akt pathway plays an important role in mediating the AC-induced protective effects, and restoration of mitochondrial damage in NSCs injured by OGD (Fig. 5)^15^.

Neurotoxicity is mediated by elevation of cytosolic Ca^2+^, and acute neural cell death correlates with the absolute amount of intracellular calcium^16,17^. Mitochondria are a major calcium-regulating organelle and mitochondrial calcium uptake and release mechanisms are key regulators of cell life or death^18,19^. Calcium overload in mitochondria and subsequent dysfunction leads to cell death after ischemic and traumatic brain injury^20,21^. The MPT pore is located in the inner mitochondrial membrane and allows solutes with molecular masses of up to 1500 Da to enter or exit the mitochondrial matrix^14^. Activation of the MPT pore is the most frequently observed consequence of extensive Ca^2+^ accumulation in the mitochondria^20^. Opening of the MPT pore results in osmotic swelling of the mitochondrial matrix that is associated with a decrease in membrane potential and release of cytochrome *c* with other apoptotic factors^14,20^. Elevated Ca^2+^ in mitochondria is thought to be a critical determinant of whether cells undergo oxidative stress-induced apoptosis^22^. Via its calcium inhibitory properties, amlodipine, a calcium channel blocker used as an antihypertensive drug^23^, can block calcium influx and reduce calcium storage in cells. Similar to our findings, a previous study reported that administration of azelnidipine, a L-type calcium antagonist, reduced not only cellular Ca^2+^ accumulation but also mitochondrial Ca^2+^ accumulation in renal cells^24^. In addition to stabilizing cellular and mitochondrial Ca^2+^ homeostasis, azelnidipine protects renal cells from apoptosis induced by hypoxic injury through inhibition of the MPT, cytochrome *c* release, and downstream signaling cascades. Amlodipine is also an L-type calcium antagonist^25^. Therefore, we hypothesized that AC would inhibit intracellular calcium influx and reduce Ca^2+^ accumulation in both the cellular cytoplasm and mitochondria of NSCs. This reduction in mitochondrial Ca^2+^ accumulation could potentially inhibit MPT pore opening, thereby preventing cytochrome *c* release, the downstream cascade, and, apoptosis of NSCs, as we demonstrated in this study.

We found that AC effectively enhanced the expression of PI3K pathway proteins, ERK, and anti-apoptotic protein Bcl-2 in OGD-injured NSCs. Similar to our findings, a previous study showed that amlodipine markedly activated the PI3K/Akt pathway in neonatal rat cardiac muscle^4^. It is well documented that the PI3K/Akt signaling pathway plays a survival role and is involved in neuroprotection by increasing levels of downstream survival-related proteins including phosphorylated Akt, phosphorylated GSK-3β, and Bcl-2, and by decreasing levels of apoptosis-related proteins including cytosolic cytochrome *c* and cleaved caspase 3^26–30^. A previous study also reported that pretreatment of human umbilical venous endothelial cells with amlodipine resulted in an increase in the Bcl-2/Bax ratio^31^. Bcl-2-family proteins can modulate intracellular Ca^2+^ transport systems at the endoplasmic reticulum, mitochondria, and plasma membrane^32^. In addition, Bcl-2-family proteins finely regulate mitochondrial calcium uptake and can maximize the positive effects of Ca^2+^ on mitochondrial bioenergetics while protecting the mitochondria from Ca^2+^ overload leading to cell death^32^. A previous study reported that Bcl-2 proteins reduce Ca^2+^ at the endoplasmic reticulum, leading to reduction of mitochondrial calcium and consequently, inhibition of cell apoptosis^19^.

We also observed in the present study that AC upregulated the expression of biogenesis-related proteins. Both PGC-1 and NRF-1 participate in mitochondrial biogenesis and respiration^33,34^. Phosphorylated dynamin-related protein (Drp) 1 reduces mitochondrial fission and promotes cell survival^35^. Mfn1 and 2 play important roles in mitochondrial fusion, and disruption of mitochondrial fusion results in mitochondrial heterogeneity and dysfunction^36^.

**Figure 6:**
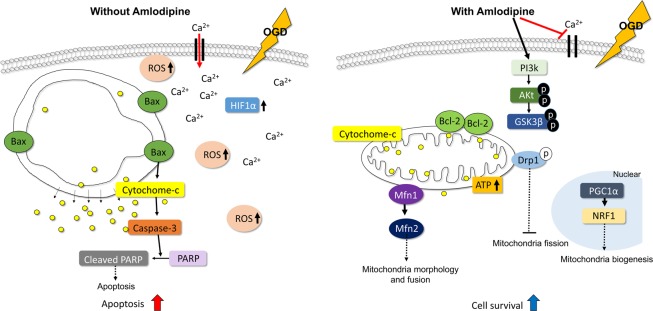
Summary of the effects of AC on NSCs under OGD. OGD disrupts mitochondria in NSCs, but AC can protect mitochondria against OGD through diverse mechanisms including inhibition of calcium influx, activation of the PI3K/Akt pathway, and mitochondria biogenesis.

Based on our results, we propose the following mechanism for the protective effects of AC in NSCs under OGD (Fig. 6). The mechanisms that were not established in this study, were referenced from above studies^33–36^. Under OGD, extracellular calcium influx occurs in NSCs with an increase in ROS and hypoxia/ischemia related protein HIF-1α. This process causes mitochondrial structural changes and activates the apoptosis-related protein Bax in the mitochondrial membrane. Mitochondrial cytochrome *c* is then released from the mitochondria to the cytosol and this process increases caspase-3 activity. Ultimately, caspase-3 induces cleavage of PARP and cell apoptosis. In contrast, when NSCs under OGD are treated with AC, calcium influx is blocked and the PI3K cell survival signaling pathway is upregulated. In addition, the survival-related protein Bcl-2, which is activated by AC, may contribute to the maintenance of the mitochondrial membrane potential and inhibit release of mitochondrial cytochrome *c*. Furthermore, AC increases the expression of mitochondrial biogenesis-related proteins such as mitofusin (Mfn) which may serve to maintain mitochondrial morphology and function in NSCs under OGD. Together, these findings indicate that AC protects NSCs from OGD and further oxidative damage and promotes cell survival via mitochondrial protection and inhibition of apoptosis.

The present study had several limitations. First, because this study was an *in vitro* study, the neuroprotective effects of AC need be re-confirmed in experimental animal models. Second, this study was conducted with AC only, which limits the generalizability of our findings. Therefore, neuroprotective effects of other amlodipine salt formulations should be investigated under the same conditions. However, considering our previous report that amlodipine besylate and AC have similar neuroprotective effects^5^, it is likely that other amlodipine salts might have similar protective effects.

In conclusion, our results provide evidence that AC protects OGD-injured NSCs by inhibiting cellular and mitochondrial calcium influx, activating the PI3K pathway, and enhancing the expression of mitochondrial biogenesis-related proteins. In addition, AC has a preservative effect on mitochondrial structure and the functions of NSCs injured by OGD. The results of the present study provide insight into the mechanisms underlying the protective effects of amlodipine on NSCs; namely, AC protect NSCs from OGD by protecting mitochondria.

## Methods

### Materials

AC was a kind gift from Hanmi Pharmaceutical Corporation (Seoul, South Korea). AC was dissolved in dimethyl sulfoxide with a final concentration in culture medium of 1% (vol/vol).

### Culture of neural stem cells and production of a hypoxic environment

All procedures involving animals were performed in accordance with the Hanyang University guidelines for the care and use of laboratory animals and were approved by the Institutional Animal Care and Use Committee (IACUC) of Hanyang University. Every effort was made to minimize the number of animals used and to limit animal suffering. Each animal was used only once.

NSCs were isolated from rodent embryonic brains, cultured, and expanded. NSC culture was performed as described previously^37–40^. Briefly, rat embryos were decapitated at embryonic day 13 (E13). Brains were rapidly removed and placed in a petri dish half full of ice-cold Hank’s balanced salt solution (HBSS; 137 mM NaCl, 5.4 mM KCl, 0.3 mM Na_2_HPO_4_, 0.4 mM KH_2_PO_4_, 5.6 mM glucose, and 2.5 mM HEPES; GIBCO BRL, Grand Island, NY, USA). Single cells were dissociated from the whole cerebral cortex, lateral ganglionic eminence, and ventral midbrain of the fetal rats. The resulting cells were plated at a density of 2 × 10^4^ cells/cm^2^ on culture dishes pre-coated with poly-L-ornithine/fibronectin in Ca^2+^/Mg^2+^-free phosphate-buffered saline (PBS; GIBCO) and cultured in N2 medium (Dulbecco’s Modified Eagle’s Medium/Nutrient Mixture F-12, 25 mg/L insulin, 100 mg/L transferrin, 30 nM selenite, 100 μM putrescine, 20 nM progesterone, 0.2 mM ascorbic acid, 2 nM L-glutamine, 8.6 mM D(+) glucose, and 20 nM NaHCO_3_; Sigma, St. Louis, MO, USA) supplemented with basic fibroblast growth factor (BFGF; 10 ng/ml, R&D Systems, Minneapolis, MN, USA). Cultures were maintained at 37 °C under a humidified 5% CO_2_ atmosphere for 4–6 days.

OGD was achieved in a Forma anaerobic chamber (Thermo Fisher Scientific, Waltham, MA USA). A gas mixture containing CO_2_ (5%), O_2_ (0.2%), and N_2_ (94.8%) was flushed through the chamber for 1, 2, 3, 4, 6, 8, and 24 h. This procedure maintained a non-fluctuating hypoxic environment below 1% O_2_. We measured hypoxic-inducible factor-1alpha (HIF-1α) (which is known to be induced by OGD) using western blotting to determine whether NSCs were affected by OGD delivered by this procedure.

To evaluate the effect of AC on NSCs, cells were treated with several concentrations of AC alone for 1 and 8 h. Finally, to measure the protective effect of AC against OGD, we treated NSCs with several concentrations of AC under OGD for 1 and 8 h, as described above.

To investigate whether the PI3K/Akt pathway plays a critical role in mediating the neuroprotection afforded by AC against OGD in NSCs, we also treated NSCs with 10 μM LY294002, a PI3K inhibitor, 30 min before treatment with 1 μM AC in the background of OGD. Cell viability and expression of proteins were assessed using TBS and western blot, respectively. NSCs were separated into 4 groups as follows: control (group 1), 1 or 8 h OGD alone (group 2), 1 or 8 h OGD + 1 μM AC (group 3), 10 μM LY294002 + 1 or 8 h OGD + 1 μM AC (group 4).

### Trypan blue staining and the lactate dehydrogenase (LDH) release assay to measure cell viability

For trypan blue staining, 10 μl aliquots of cells were incubated with 10 μl of trypan blue solution for 2 m. Unstained live cells were counted with a hemocytometer. A colorimetric assay kit (Roche Boehringer–Mannheim, IN, USA) was used to quantify LDH released from cultured NSCs according to the manufacturer’s instructions. Cell viability was assessed using an ELISA plate reader (Synergy H1 Hybrid reader, BioTek Instruments, Winooski, VT, USA) at 490 nm with a reference wavelength of 690 nm. All results were normalized to the OD of an identical well without cells^41^.

### DAPI and TUNEL staining to evaluate apoptosis

NSCs were seeded on collagen-coated 13 mm diameter glass cover slips and treated with AC without exposure to OGD, without AC but with exposure to OGD for 8 h, or with exposure to OGD in the presence of AC (0.1 and 1 μM) for 8 h. Cells were then rinsed twice with PBS, air-dried, and fixed with 4% paraformaldehyde in PBS for 1 h at room temperature.

Apoptotic cell death was identified by terminal deoxynucleotidyl transferase-mediated deoxyuridine triphosphate nick end labeling (TUNEL) (Roche Boehringer–Mannheim, IN, USA). To monitor intact, condensed, and fragmented nuclei, TUNEL-stained cells were counterstained with 4’,6-diamidino-2-phenylindole (DAPI, Vector Laboratories, Burlingame, CA, USA) for 20 m, washed several times with PBS, and mounted on glass slides with mounting medium (Merck, Kenilworth, NJ, USA). Cells were then observed under an Olympus Bx53 microscope (Olympus, Tokyo, Japan).

### Annexin V and propidium iodide (PI) staining and detection

Annexin V/PI staining was measured using a FITC Annexin V Apoptosis Detection Kit I (Beckton Dickinson, Franklin Lakes, NJ, USA) according to the manufacturer’s instructions. Briefly, cortical neurons were exposed to OGD with AC (0.1 and 1 µM) and incubated for 8 h at 37 °C. Cells were then rinsed twice with cold PBS and resuspended in 1× Binding Buffer. They were transferred (100 μl of the solution; 1 × 10^5^ cells) to a 1.5 ml culture tube followed by application of 5 μl of FITC Annexin V and 10 μl PI. Samples were gently mixed and incubated for 15 m at room temperature in the dark. Finally, 400 μl of 1× Binding Buffer was added to each tube and samples were analyzed by flow cytometry (Accuri C6 Flow cytometer, BD Biosciences). Data were acquired and analyzed with BD Accuri C6 software.

### Colony-forming unit assay

Proliferation of NSCs was measured via a colony-forming unit (CFU) assay. Approximately 0.5 × 10^4^ cells were seeded in a 60-mm grid plate and treated with OGD and AC (0.1 and 1 µM) for 8 h. Cells were then washed with DPBS, and the culture medium was changed. After 14 days, cells were washed again with DPBS and stained with 0.5% crystal violet (Sigma) in methanol for 30 m at room temperature. After staining, plates were washed with DPBS and allowed to dry. Colonies were counted under a dissecting microscope and colonies less than 2 mm in diameter or faintly stained were excluded.

### Migration assays

Cell migration assays were performed using a QCM 24-well colorimetric cell migration assay kit (Chemicon, Temecula, CA, USA) according to the manufacturer’s instructions. Briefly, OGD and AC (0.1 and 1 µM) were added into the upper chamber of the plate followed by incubation for 24 h at 37 °C. Cells that had migrated through the membrane were stained, and the number of migrated cells was determined by measuring the absorbance at 560 nm using a spectrophotometric plate reader (Synergy H1, BioTek Instruments)^42^.

### BrdU cell proliferation assay

NSCs were incubated in BrdU-labeling medium (10 μM BrdU) for 5 h, and cell proliferation was measured using a BrdU Labeling and Detection Kit (Roche Boehringer–Mannheim, IN, USA) according to the manufacturer’s instructions. Cell proliferation was assessed using an ELISA plate reader (Synergy H1, BioTek Instruments) at 370 nm with a reference wavelength of 492 nm. All results were normalized to the OD of an identical well without cells.

### Electron microscopy

NSCs (1 × 10^6^) were seeded on 150 mm dishes and exposed to OGD and AC (0.1 and 1 µM) for 8 h. Cells were then washed twice with PBS and fixed with EM fixing buffer for 1 h at room temperature. Twenty-four hours after MPP + (1-methyl-4-phenylpyridinium) treatment, cells were washed with DMEM and fixed in 0.1 M cacodylate buffer (pH 7.0, TED PELLA INC., Redding, CA, USA) containing 2% paraformaldehyde (Merck) and 0.5% glutaraldehyde (EMS, Hatfield, PA, USA) at 37 °C for 15 m, after which fixation was allowed to proceed at room temperature. Cells were washed with the same buffer three times, post-fixed with 1% osmium tetroxide (Heraeus, Hanau, Germany) for 30 m, and EM bloc-stained with 0.2% uranyl acetate (EMS) solution for 1 h. Subsequently, cells were dehydrated through an ascending ethanol series and embedded in an Epon mixture. Thin sections of 80 nm were cut using a Reichert-Jung Ultracut E ultramicrotome (Leica, Wetzlar, Germany) and mounted on a 200-mesh grid. Electron microscopy observation was performed with a Hitachi H-7500 transmission electron microscope (Hitachi, Ltd., Tokyo, Japan) with 80 kV acceleration voltage.

### Determination of free radical production

To measure free radical production, NSCs were exposed to OGD for 30 m and incubated with the fluorescent probe 2′,7′-dichlorodihydrofluorescein diacetate (H_2_DCF-DA) (Molecular Probes Inc., Eugene, OR, USA) for 15 m. Then, NSCs treated with different concentrations of AC were exposed to OGD for 8 h followed by sample reoxygenation for 4 h. After incubation with 10 µM H_2_DCF-DA at 37 °C for 15 m, cells were washed with PBS three times. H_2_DCF-DA freely crosses cell membranes and is hydrolyzed by cellular esterases to 2′,7′-dichlorodihydrofluorescein (H_2_DCF), which is oxidized to 2′7′-dichlorofluorescein (DCF), which fluoresces in the presence of peroxides. Therefore, DCF fluorescence indicates the level of intracellular hydrogen peroxide, not superoxide. Accumulation of DCF in cells was assessed using an ELISA plate reader (Synergy H1 reader, BioTek Instruments) by measuring fluorescence (excitation 488 nm; emission 530 nm). All results were normalized to the OD of an identical well without cells.

### Mitochondrial membrane potential assay

NSCs were seeded at a density of 5 × 10^5^/ml per well of a 96-well culture plate. Cells were exposed to OGD with AC (0.1 and 1 µM) and incubated for 8 h at 37 °C. Mitochondrial membrane potential was measured using a JC-1 Mitochondrial Membrane Potential Assay Kit (Abnova, Taipei Taiwan) according to the manufacturer’s instructions. Mitochondrial membrane potential was assessed using an ELISA plate reader (Synergy H1 reader, BioTek Instruments). Apoptotic or unhealthy cells with mainly JC-1 monomers were detected with settings designed to detect FITC (excitation/emission = 485/535 nm).

### ATP assay

ATP concentrations were measured using an ATP assay kit (Abcam, Cambridge, UK) according to the manufacturer’s instructions and fluorimetrically assessed using an ELISA plate reader (Synergy H1 reader, BioTek Instruments). ATP concentration was detected with an excitation/emission setting of 535/587 nm.

### Determination of Ca^2+^ level

Intracellular calcium levels in NSCs was measured via a Fura 2-AM. Approximately 1–2 × 105 cells were seeded in a 24 well plate and treated with OGD and AC (0.1 and 1 µM) for 4 h. Cells were incubated with 10 mM Fura-2-AM (Sigma) for 1 h at 25–37 °C, in Hank’s balanced salt solution (HBSS), HEPES buffer saline (87 mM NaCl, 50 mM KCl, 1 mM MgCl2, 12 mM HEPES, 10 mM Glucose and 5 mM CaCl2 (pH 7.4). After 1 h of incubation, cells were washed with HEPES buffer saline for 30 min at room temperature. Fura-2 loaded cells were illuminated with dual excitation wave lengths of 340 nm and 380 nm, while capturing the emission signal at 510 nm with the help of appropriate excitation and emission filters (Synergy H1, BioTek Instruments).

NSCs were treated for 8 h, the culture medium was replaced with new medium containing 5 μM Fluo-4 AM (Life Technologies, Carlsbad, IL, USA), and cells were incubated at 37 °C for 1 h. Cells were washed in PBS and incubated for 30 m to allow complete de-esterification of intracellular acetomethyl (AM) esters for fluorescence analysis. Accumulation of fluo-4AM in cells was assessed using an ELISA plate reader (Synergy H1 reader, BioTek Instruments) by measuring fluorescence (excitation 505 nm; emission 530 nm).

Fluo-4 AM intensity was quantified within a region of interest (ROI) for each cell and expressed as the relative change in fluorescence: Δ*F*/*F*_0_ = (*F* − *F*_0_)/*F*_0_, where *F*_0_ is the fluorescence level at the start of the experiment after subtracting the background fluorescence. Peak amplitudes of Δ*F*/*F*_0_ were determined on stimulation and cumulative increases in Δ*F*/*F*_0_ were calculated over a 420 s post-stimulus time period using Microsoft Excel.

### Western blot analyses

Levels of phosphatidylinositol 3-kinase (PI3K), phosphorylated Akt (pAkt) (Ser473), phosphorylated glycogen synthase kinase-3β (pGSK-3β) (Ser9), phospho-p44/42 MARK (Erk1/2), HIF-1α, Ki67, phosphor-p38 (Thr180/Thr182), peroxisome proliferator-activated receptor gamma coactivator 1-alpha (PGC1 α), nuclear respiratory factor 1 (NRF-1), dynamin-related protein 1 (Drp1), mitofusin 1 (Mfn 1), mitofusin 2 (Mfn 2), Bcl-2, Bax, cytochrome-C and cleaved PARP were analyzed by western blotting^43^. Western blotting was performed immediately after treatment for 1 or 8 h. Briefly, 5 × 10^6^ cells were washed twice in cold PBS, incubated for 30 m on ice in lysis buffer [RIPA II cell lysis buffer 1× with Triton, without EDTA; 1 mM phenylmethylsulfonyl fluoride (PMSF); 1 mM sodium fluoride (NaF); 1 mM sodium orthovanadate (Na_3_VO_4_); and 0.5% protease inhibitor cocktail 1x]. Next, cells were sonicated (Sonoplus, Bandelin Electronics, Berlin, Germany) and incubated for 30 m on ice. To evaluate cytosolic and mitochondria cytochrome *c* levels, mitochondrial and cytosolic fractions were isolated using the Mitochondria/Cytosol Fractionation Kit (Abcam, UK) according to the manufacturer’s instructions. Briefly, after OGD for 8 h with different concentrations of AC treatment, NSCs were harvested, washed once with ice-cold PBS, and resuspended in 1.0 mL of 1× cytosol extraction buffer mix containing dithiothreitol (DTT) and protease inhibitors. After a 10-m incubation on ice, the cell suspension was sonicated (Sonoplus) 5–10 times on ice. Samples were centrifuged at 3,000 rpm at 4 °C for 10 m. Supernatants were centrifuged again at 13,000 rpm for 30 m to separate the mitochondrial fraction (pellet) and the cytosolic fraction (supernatant). The mitochondria pellet was washed once with the isolation buffer, and then lysed in mitochondrial extraction buffer containing DTT and protease inhibitors. Samples containing equal amounts (30 μg) of protein were resolved by 10% sodium dodecyl sulfate-polyacrylamide gel electrophoresis (SDS-PAGE) and transferred to nitrocellulose membranes (Amersham Pharmacia Biotech, Buckinghamshire, UK). Membranes were blocked with 5% skim milk and then incubated with specific primary antibodies. The antibodies used were as follows: anti-p85α PI3K (1:1000, Millipore), anti-PI3K p110 (1:1000, Cell signaling, Beverly, MA, USA), anti-pAkt (1:500, Cell Signaling), anti-Akt (1:1000, Cell Signaling), anti-pGSK-3β (Ser9) (1:500, Cell Signaling, Beverly, MA, USA), anti- p-p44/42 MARK (1:1000, Cell Signaling), anti-HIF-1 (1:1000, Santa Cruz, CA, USA), anti-Ki67 (1:200, Abcam, UK), anti-p-p38 (1:1000, Cell Signaling), anti-PGC1α (1:1000, Abcam), anti-NRF-1 (1:1000, Abcam), Drp1 (1:500, Cell Signaling), anti-Mfn 1 (5 μg/ml, Abcam), anti-Mfn 2 (5 μg/ml, Abcam), anti-Bcl-2 (1:500, Abcam), anti-Bax (1:1000, Abcam), anti-cytochrome *c* (1:200, Cell Signaling), and anti-PARP (1:400, Cell Signaling). Membranes were washed with Tris-buffered saline containing 0.1% Tween-20 (TBST), and then processed using HRP-conjugated anti-rabbit antibody or anti-mouse antibody (Amersham Pharmacia Biotech, Piscataway, NJ, USA) followed by ECL detection (Amersham Pharmacia Biotech). Blots were quantified with an image analyzer (GE Healthcare, ImageQuant LAS 4000).

### Statistical analysis

All data are presented as means ± standard deviations of five or more independent experiments. Viabilities of different treatment groups were compared with Tukey’s test after one-way analysis of variance (ANOVA). Levels of apoptosis and free radicals in different treatment groups and the western blotting results were compared using Tukey’s test after two-way ANOVA. P-values less than 0.05 were considered statistically significant.

## Supplementary information

Supplementary information

## Data Availability

All data generated or analysed during this study are included in this published article (and its Supplementary Information Files).

## References

[1] Zhao L-P (2013). Oxygen glucose deprivation (OGD)/re-oxygenation-induced *in vitro* neuronal cell death involves mitochondrial cyclophilin-D/P53 signaling axis. Neurochem. Res..

[2] Hsieh, Y. S., Kwon, S., Lee, H. S. & Seol, G. H. Linalyl acetate prevents hypertension-related ischemic injury. *PLoS ONE* **13** (2018). 10.1371/journal.pone.0198082 PMC5969747 29799836

[3] Shakoor A (2014). Effect of L-type calcium channel blocker (amlodipine) on myocardial iron deposition in patients with thalassaemia with moderate-to-severe myocardial iron deposition: protocol for a randomised, controlled trial. BMJ Open.

[4] Li X-Q (2009). Amlodipine inhibits TNF-alpha production and attenuates cardiac dysfunction induced by lipopolysaccharide involving PI3K/Akt pathway. Int. Immunopharmacol..

[5] Lee YJ, Park H-H, Koh S-H, Choi N-Y, Lee K-Y (2011). Amlodipine besylate and amlodipine camsylate prevent cortical neuronal cell death induced by oxidative stress. J. Neurochem..

[6] Choi N-Y (2014). Neuroprotective effects of amlodipine besylate and benidipine hydrochloride on oxidative stress-injured neural stem cells. Brain Res..

[7] Yamagata K, Ichinose S, Tagami M (2004). Amlodipine and carvedilol prevent cytotoxicity in cortical neurons isolated from stroke-prone spontaneously hypertensive rats. Hypertens. Res. Off. J. Jpn. Soc. Hypertens..

[8] Mogi M (2006). Amlodipine Treatment Reduces Stroke Size in Apolipoprotein E–Deficient Mice. Am. J. Hypertens..

[9] Siesjö BK, Bengtsson F (1989). Calcium Fluxes, Calcium Antagonists, and Calcium-Related Pathology in Brain Ischemia, Hypoglycemia, and Spreading Depression: A Unifying Hypothesis. J. Cereb. Blood Flow Metab..

[10] Lukic-Panin V (2007). Prevention of neuronal damage by calcium channel blockers with antioxidative effects after transient focal ischemia in rats. Brain Res..

[11] Kawai H (2011). Protection against ischemic stroke damage by synergistic treatment with amlodipine plus atorvastatin in Zucker metabolic rat. Brain Res..

[12] Thored P (2006). Persistent production of neurons from adult brain stem cells during recovery after stroke. Stem Cells Dayt. Ohio.

[13] Pivovarova NB, Andrews SB (2010). Calcium-dependent mitochondrial function and dysfunction in neurons. FEBS J..

[14] Norenberg MD, Rao KVR (2007). The mitochondrial permeability transition in neurologic disease. Neurochem. Int..

[15] Trotta AP, Chipuk JE (2017). Mitochondrial dynamics as regulators of cancer biology. Cell. Mol. Life Sci. CMLS.

[16] Hartley DM, Kurth MC, Bjerkness L, Weiss JH, Choi DW (1993). Glutamate receptor-induced 45Ca2+ accumulation in cortical cell culture correlates with subsequent neuronal degeneration. J. Neurosci. Off. J. Soc. Neurosci..

[17] Eimerl S, Schramm M (1994). The quantity of calcium that appears to induce neuronal death. J. Neurochem..

[18] Friel DD (2000). Mitochondria as regulators of stimulus-evoked calcium signals in neurons. Cell Calcium.

[19] Giacomello M, Drago I, Pizzo P, Pozzan T (2007). Mitochondrial Ca^2+^ as a key regulator of cell life and death. Cell Death Differ..

[20] Starkov AA, Chinopoulos C, Fiskum G (2004). Mitochondrial calcium and oxidative stress as mediators of ischemic brain injury. Cell Calcium.

[21] Friberg H, Wieloch T (2002). Mitochondrial permeability transition in acute neurodegeneration. Biochimie.

[22] Baumgartner HK (2009). Calcium Elevation in Mitochondria Is the Main Ca2+ Requirement for Mitochondrial Permeability Transition Pore (mPTP) Opening. J. Biol. Chem..

[23] Ishimitsu T (1999). Amlodipine, a long-acting calcium channel blocker, attenuates morning blood pressure rise in hypertensive patients. Clin. Exp. Pharmacol. Physiol..

[24] Tanaka T (2004). Blockade of Calcium Influx through L-Type Calcium Channels Attenuates Mitochondrial Injury and Apoptosis in Hypoxic Renal Tubular Cells. J. Am. Soc. Nephrol..

[25] Kale OE, Awodele O, Ogundare TF, Ekor M (2017). Amlodipine, an L-type calcium channel blocker, protects against chlorpromazine-induced neurobehavioural deficits in mice. Fundam. Clin. Pharmacol..

[26] Wang C, Wei Z, Jiang G, Liu H (2017). Neuroprotective mechanisms of miR-124 activating PI3K/Akt signaling pathway in ischemic stroke. Exp. Ther. Med..

[27] Li Z, Gang ZZ, Shuang LX, Ann H-S, Michael C (2007). The PI3K/Akt Pathway Mediates the Neuroprotective Effect of Atorvastatin in Extending Thrombolytic Therapy After Embolic Stroke in the Rat. Arterioscler. Thromb. Vasc. Biol..

[28] Lee YJ (2009). Cilnidipine mediates a neuroprotective effect by scavenging free radicals and activating the phosphatidylinositol 3-kinase pathway. J. Neurochem..

[29] Cantley LC (2002). The phosphoinositide 3-kinase pathway. Science.

[30] Pap M, Cooper GM (2002). Role of translation initiation factor 2B in control of cell survival by the phosphatidylinositol 3-kinase/Akt/glycogen synthase kinase 3beta signaling pathway. Mol. Cell. Biol..

[31] Bian Y (2011). Amlodipine treatment prevents angiotensin II-induced human umbilical vein endothelial cell apoptosis. Arch. Med. Res..

[32] Vervliet T, Parys JB, Bultynck G (2016). Bcl-2 proteins and calcium signaling: complexity beneath the surface. Oncogene.

[33] Scarpulla RC (2011). Metabolic control of mitochondrial biogenesis through the PGC-1 family regulatory network. Biochim. Biophys. Acta.

[34] Shao D (2010). PGC-1 beta-regulated mitochondrial biogenesis and function in myotubes is mediated by NRF-1 and ERR alpha. Mitochondrion.

[35] Jahani-Asl A, Slack RS (2007). The phosphorylation state of Drp1 determines cell fate. EMBO Rep..

[36] Chen H, Chomyn A, Chan DC (2005). Disruption of Fusion Results in Mitochondrial Heterogeneity and Dysfunction. J. Biol. Chem..

[37] Currle, D. S., Hu, J. S., Kolski-Andreaco, A. & Monuki, E. S. Culture of mouse neural stem cell precursors. *J*. *Vis*. *Exp*. *JoVE* **152**, 10.3791/152 (2007). 10.3791/152 PMC2532938 18830426

[38] Choi YK (2016). Dual effects of carbon monoxide on pericytes and neurogenesis in traumatic brain injury. Nat. Med..

[39] Chojnacki A, Weiss S (2008). Production of neurons, astrocytes and oligodendrocytes from mammalian CNS stem cells. Nat. Protoc..

[40] Studer L, Tabar V, McKay RD (1998). Transplantation of expanded mesencephalic precursors leads to recovery in parkinsonian rats. Nat. Neurosci..

[41] Park H-H, Lee K-Y, Kim SH, Lee YJ, Koh S-H (2009). l-DOPA-induced neurotoxicity is reduced by the activation of the PI3K signaling pathway. Toxicology.

[42] Koh S-H, Noh MY, Cho GW, Kim KS, Kim SH (2009). Erythropoietin increases the motility of human bone marrow-multipotent stromal cells (hBM-MSCs) and enhances the production of neurotrophic factors from hBM-MSCs. Stem Cells Dev..

[43] Choi H, Choi N-Y, Lee K-Y, Lee YJ, Koh S-H (2017). Candesartan Restores the Amyloid Beta-Inhibited Proliferation of Neural Stem Cells by Activating the Phosphatidylinositol 3-Kinase Pathway. Dement. Neurocognitive Disord..

