# Supplementary material for: Mitochondria damaged by Oxygen Glucose Deprivation can be Restored through Activation of the PI3K/Akt Pathway and Inhibition of Calcium Influx by Amlodipine Camsylate
Source: Sci Rep. 2019 Oct 31;9:15717. doi: 10.1038/s41598-019-52083-y (PMC6823474; doi:10.1038/s41598-019-52083-y)
Supplement: Supplementary file 1 — Supplementary information [file 41598_2019_52083_MOESM1_ESM.docx]

**Supplementary information**

**Title:** Mitochondria damaged by Oxygen Glucose Deprivation can be Restored through Activation of the PI3K/Akt Pathway and Inhibition of Calcium Influx by Amlodipine Camsylate

Authors: Hyun-Hee Park, Myung-Hoon Han, Hojin Choi, Young Joo Lee, Jae Min Kim, Jin Hwan Cheong, Je Il Ryu, Kyu-Yong Lee, Seong-Ho Koh

**Supplementary data:**

**Figures S1 - 6**


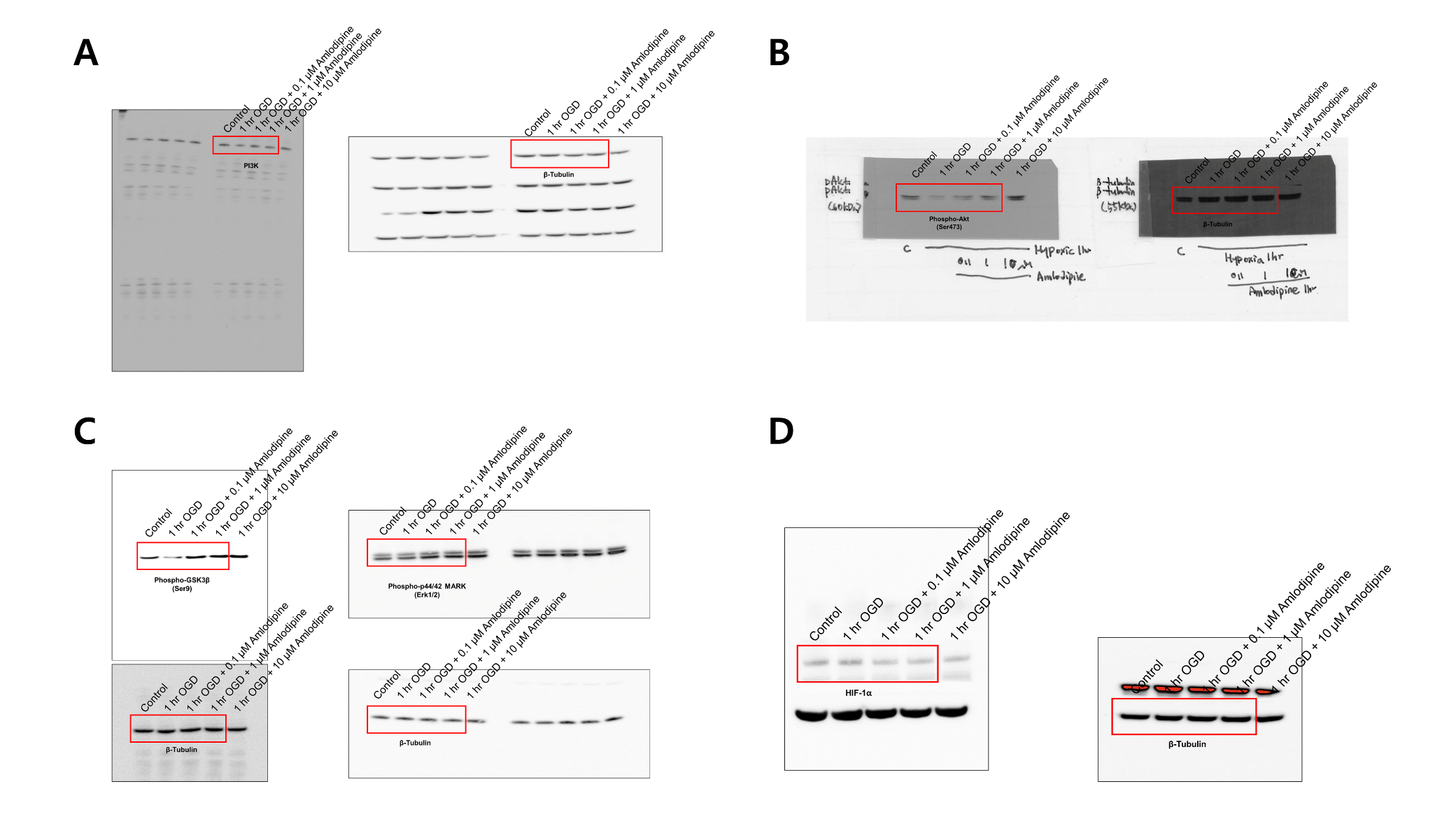


**Supplementary Fig. S1.** Full-length blots/gels of Figure 1F


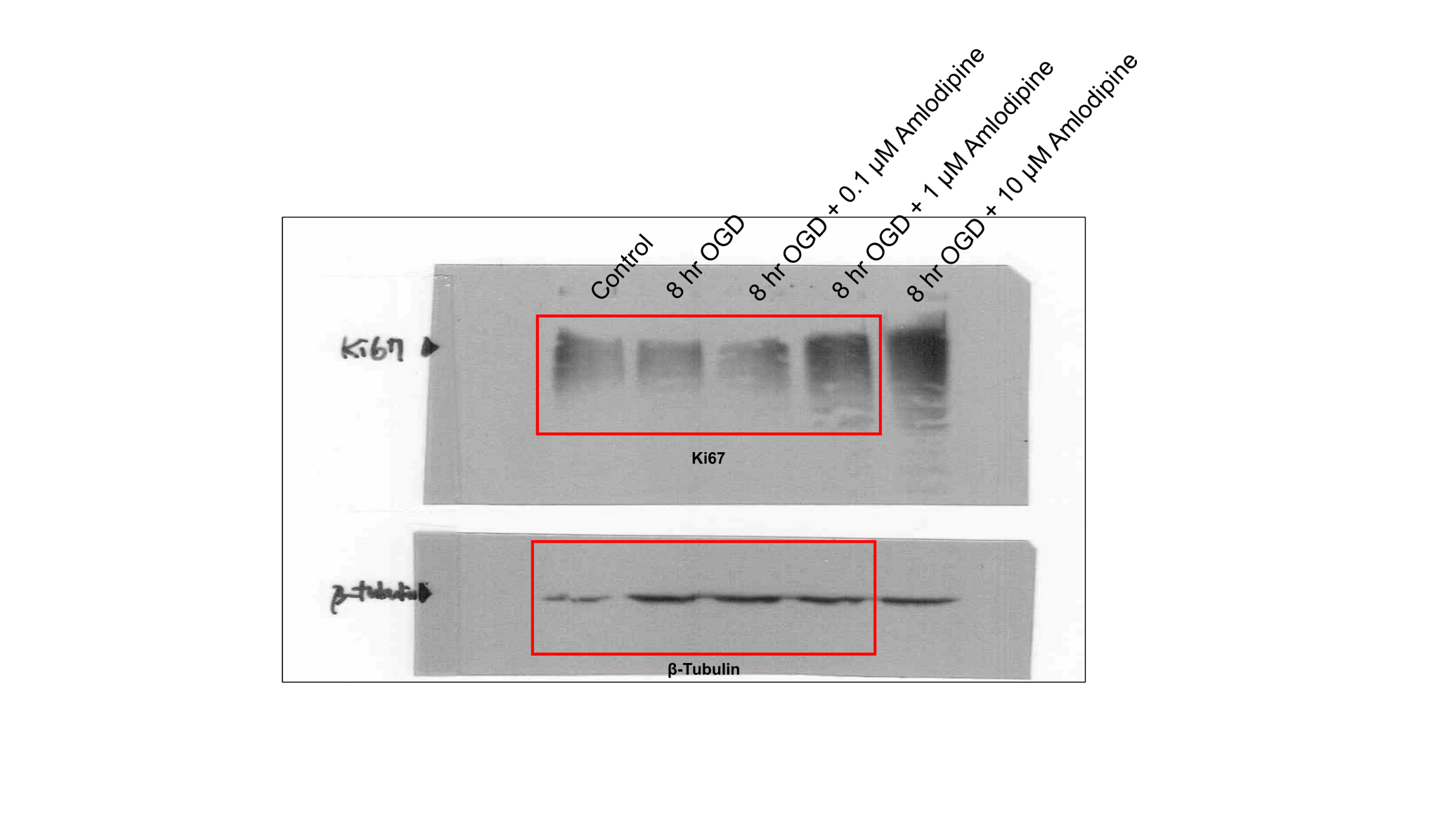


**Supplementary Fig. S2.** Full-length blots/gels of Figure 2C


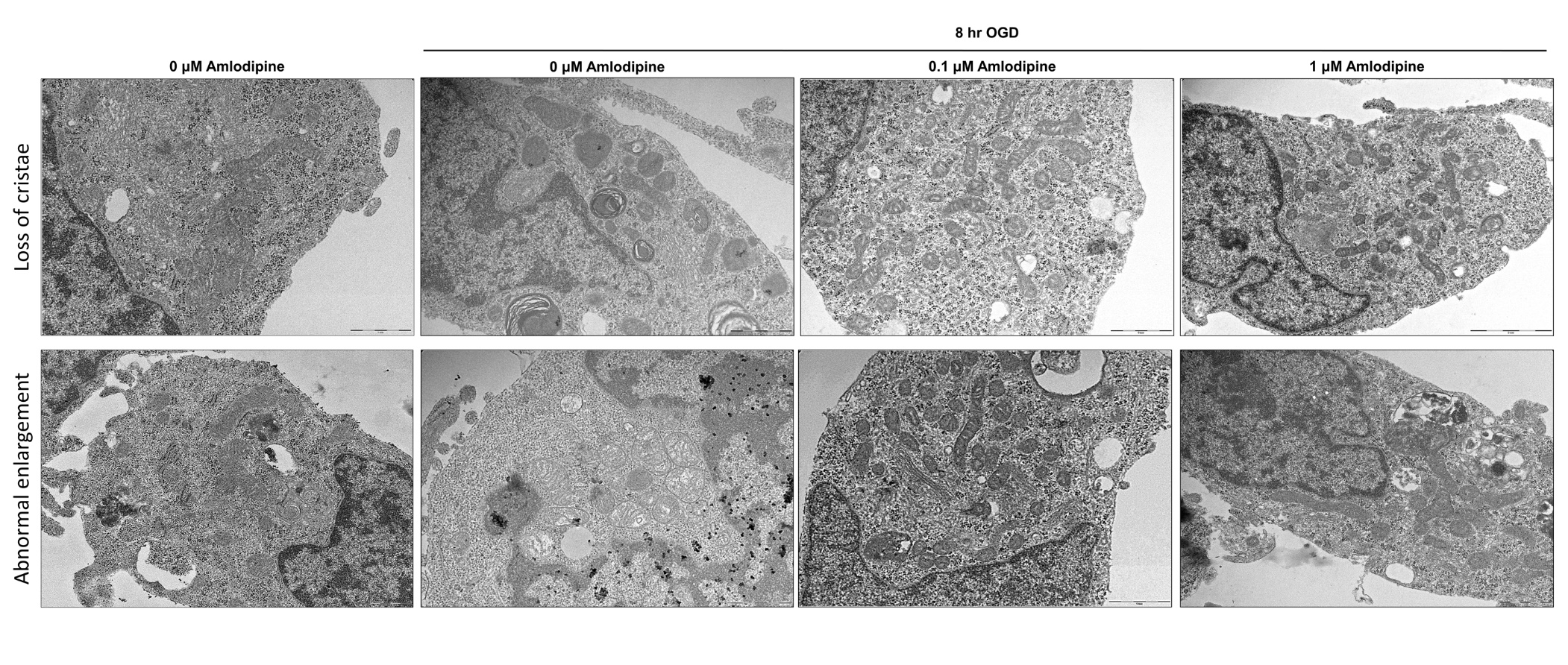


**Supplementary Fig. S3.** Effect of OGD and AC on NSC mitochondrial damage.

Representative transmission electron microscopy (TEM) image of NSCs treated with OGD only or OGD and AC showing mitochondrial damage in the form of loss of cristae (top) and abnormal elongation (bottom).


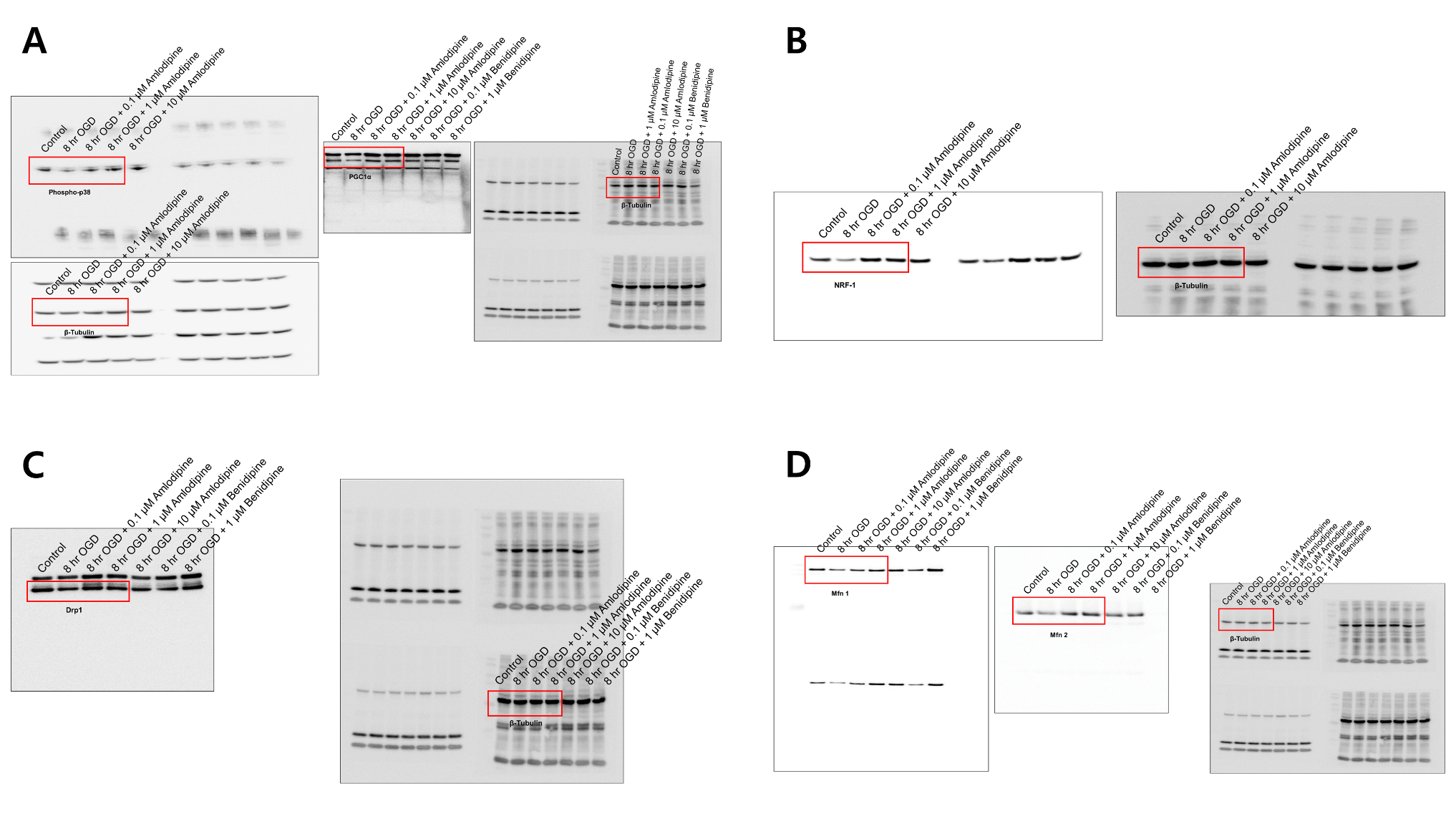


**Supplementary Fig. S4.** Full-length blots/gels of Figure 3B


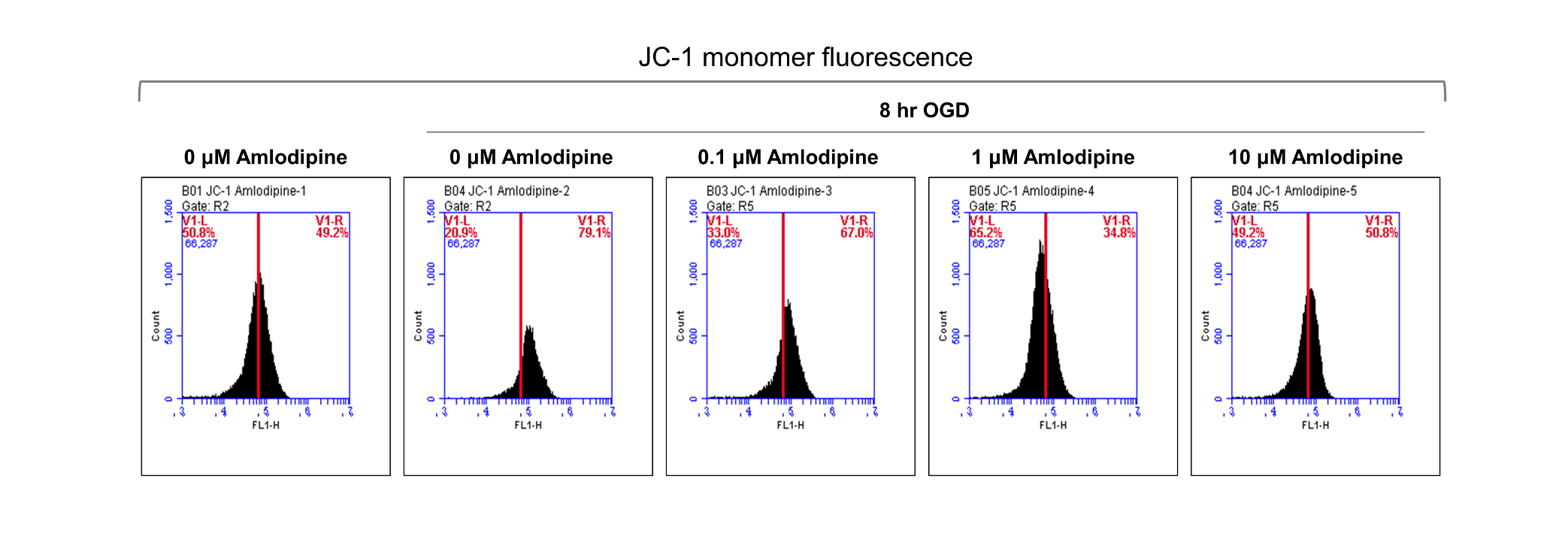


**Supplementary Fig. S5.** Effect of AC on mitochondrial membrane potential in NSCs injured by OGD.

Using FACS, lower JC-1 monomer expression was confirmed in NSCs co-treated with AC and OGD than NSCs treated with OGD alone.


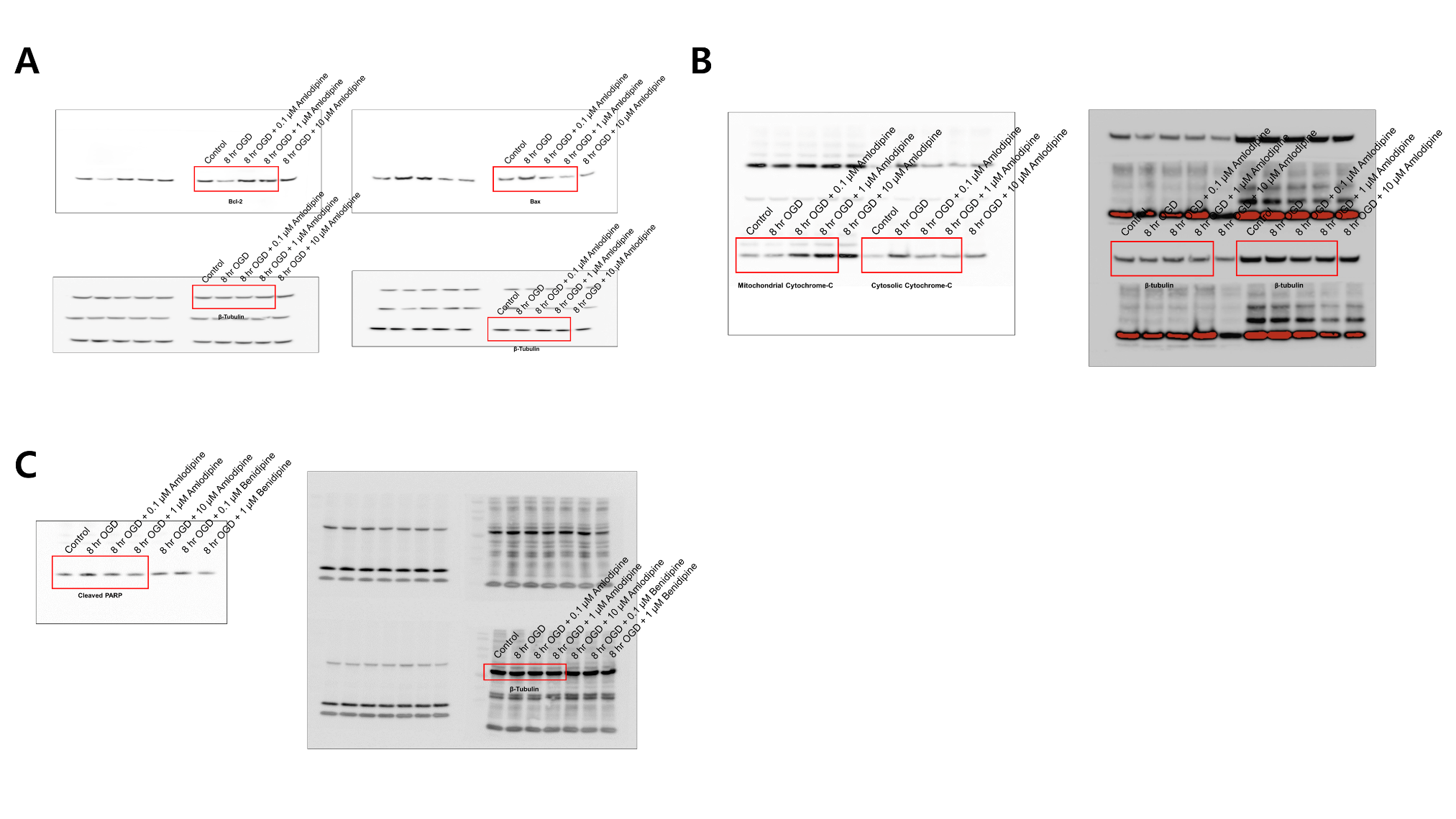


**Supplementary Fig. S6.** Full-length blots/gels of Figure 4E


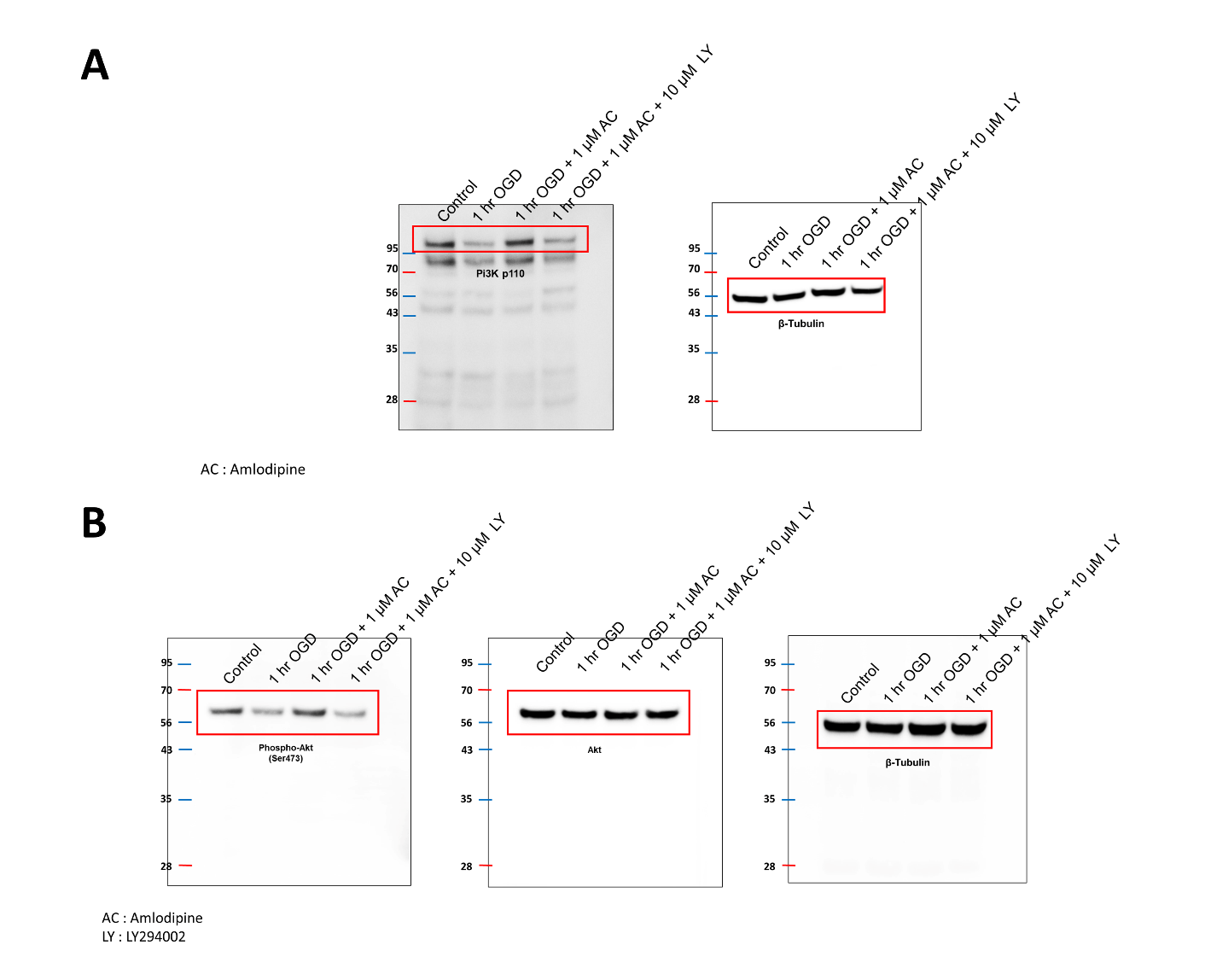


**Supplementary Fig. S7.** Full-length blots/gels of Figure 5B


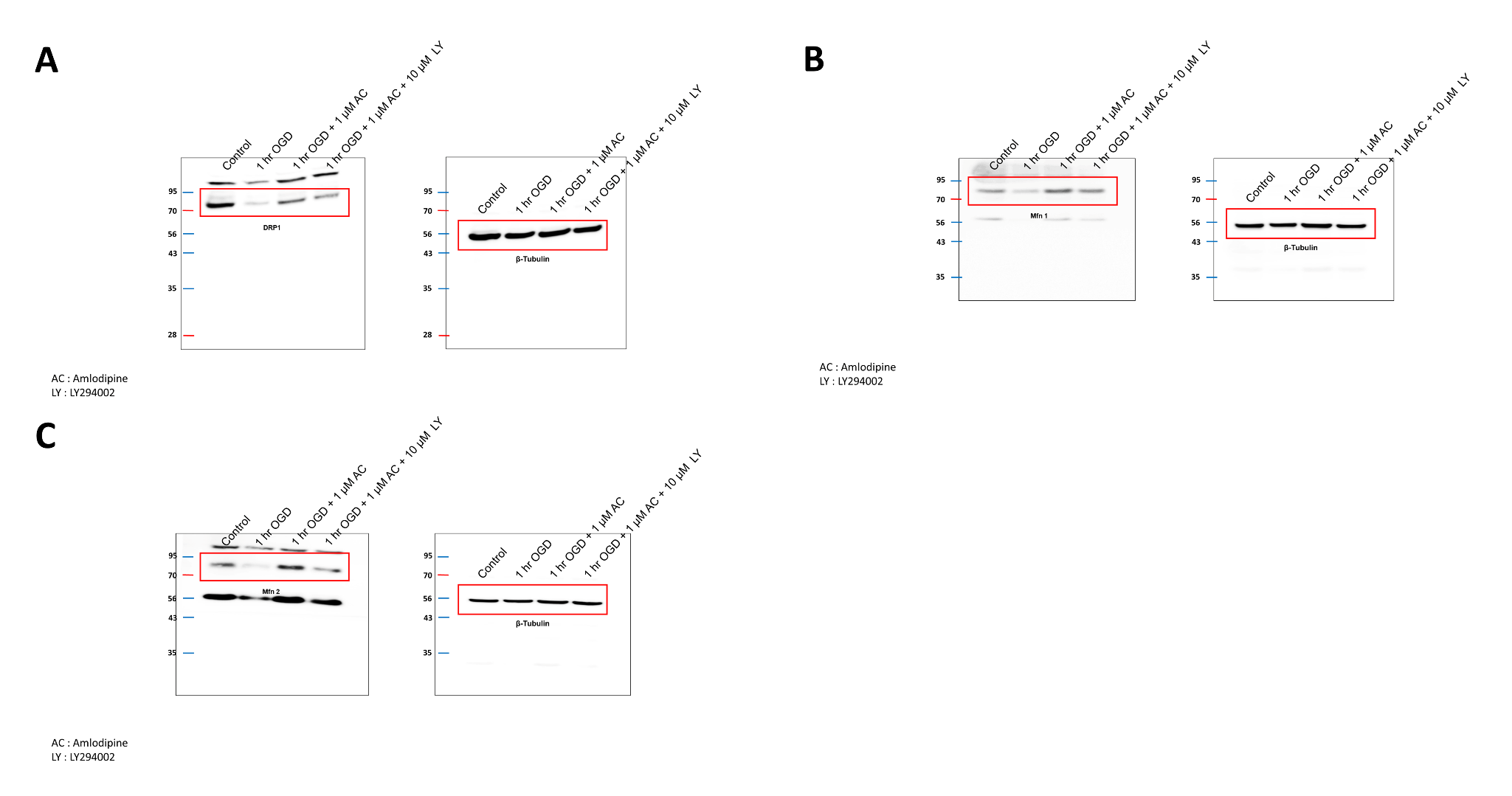


**Supplementary Fig. S8.** Full-length blots/gels of Figure 5C
